# A selective review of inhibitors of protein kinase C gamma: a neuroplasticity-related common pathway for psychiatric illness

**DOI:** 10.3389/fddev.2024.1364037

**Published:** 2024-09-13

**Authors:** Marco Grados, Mona Salehi, Aida Lotfi, Sagar Dua, Isabella Xie

**Affiliations:** ^1^ Psychiatry and Behavioral Sciences, School of Medicine, Johns Hopkins University, Baltimore, MD, United States; ^2^ Johns Hopkins University, Baltimore, MD, United States; ^3^ School of Medicine, Johns Hopkins University, Baltimore, MD, United States; ^4^ School of Health and Welfare, Jönköping University, Jönköping, Sweden; ^5^ All India Institute of Medical Sciences, New Delhi, India; ^6^ Palo Alto University, Palo Alto, CA, United States

**Keywords:** PKC isoforms, anxiety, neuroplasticity, fluoxetine (CID:62857), PKC gamma

## Abstract

Psychotropics are currently developed and marketed with a limited understanding of their mechanism of action. The notion that protein kinase C (PKC) activity is highly relevant to learning and memory function stems from experiments in the 1980s, which associated protein kinase alpha (pka) and pkc to animal models of associative learning, opening an area of exploration for psychotropic development. The PKC family consists of several isoforms, including PKC alpha, beta1, beta1, gamma, delta and epsilon among others. In particular, PKC gamma (PRKCG) is highly brain-expressed and is singled out as a candidate for modulation in psychiatric illness. With hundreds of identified substrates, PRKCG affects multiple pathways relevant for regulation of neuronal health. In this review, converging lines of evidence are presented in the context of psychotropic drug action, which point to downregulation of PKC activity as a potential common mechanism across several psychiatric disorders. Using this mechanism through more targeted psychotropic action may then be used to develop agents that further ameliorate psychiatric symptom expression. Psychotropics including fluoxetine, tricyclics, lithium, valproate, ketamine and others are explored in relation to their effect of PKC, finding that across all drugs examined, a downregulation with chronic-but not acute-use constitutes their putative effect in ameliorating symptoms. This effect is compounded by findings that suggest that PKCs, and PRKCG in particular, promote neuroplastic effects by their downregulation. This effect is in contrast to PKC activators, which have been used in neurodegenerative disorders such as Alzheimer’s disease. Cross-disorder mechanisms need to continue to be explored in neuropsychiatric illness and targeted treatments developed in turn to address treatment-resistant conditions.

## 1 Introduction

**FIGURE 1:**
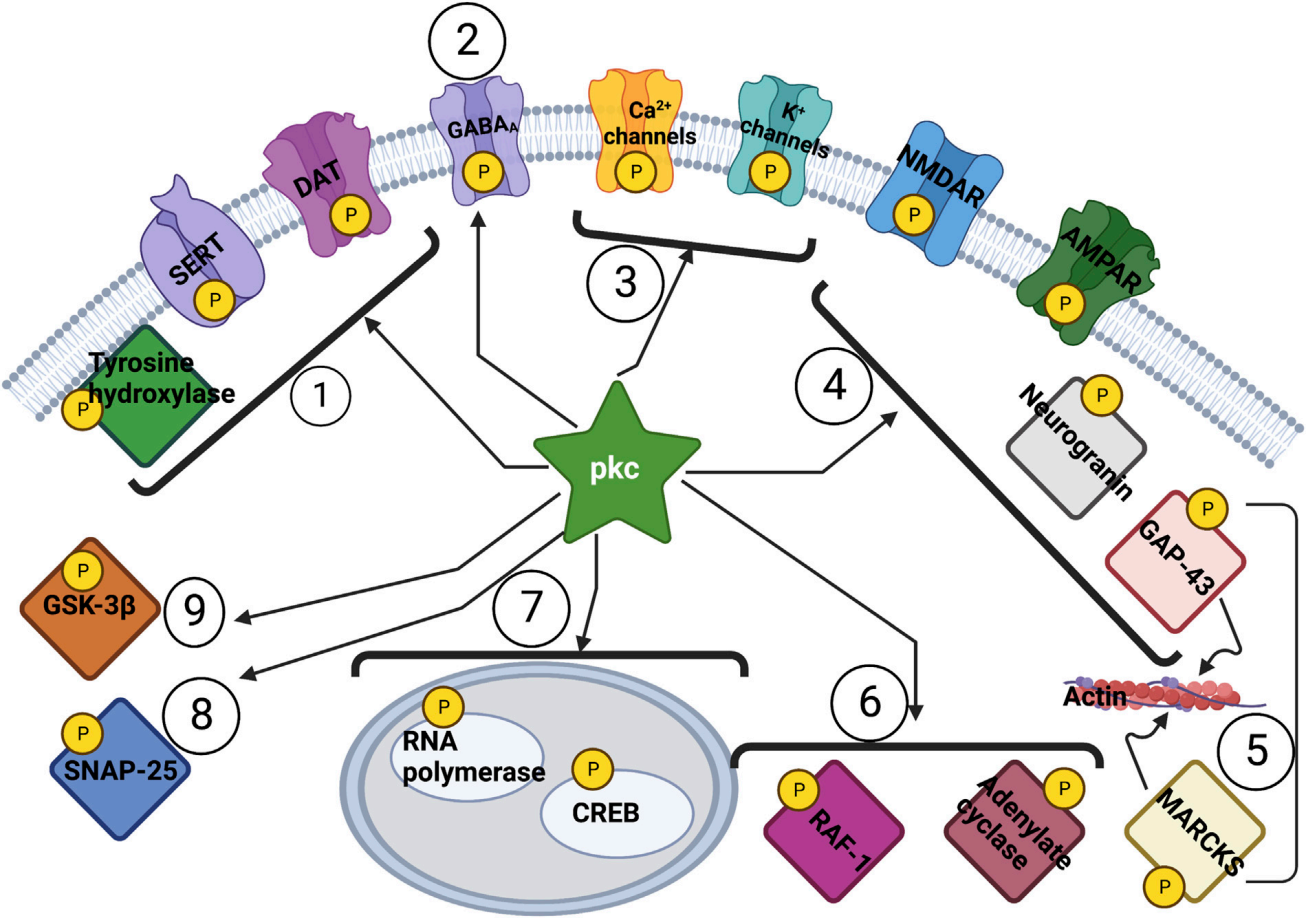
**Action of PKC across Biological Systems.** One Monoamine transport and synthesis (Ramamoorthy et al., 1998); 2 Inhibitory neurotransmission (Meier et al., 2003; Sachidanandan et al., 2017); 3 Membrane excitability (Andersen et al., 2018; Du et al., 2021; Gada and Logothetis, 2022); 4 Glutamatergic Transmission and LTP (Malinow et al., 1989; Kleschevnikov and Routtenberg, 2001; Boehm et al., 2006); 5 Calcium regulation and cytoskeletal restructuring (Keenan and Kelleher, 1998; Larsson, 2006; Long and Freeley, 2014); 6 Intracellular signaling pathways (Frings, 1993; Moughal et al., 1995); 7 Transcription (Guo and Feng, 2012; Obi et al., 2017; Wu et al., 2018); 8 Exocytosis (Morgan et al., 2005; Lian et al., 2009; Yasuda et al., 2011); 9 Glycogen metabolism and synaptic plasticity (Garzón-Niño et al., 2017; Wu et al., 2017) AMPAR: α-amino-3-hydroxy-5-methyl-4- isoxazole propionic acid receptor; CREB: cAMP regulatory element-binding protein; DAT: dopamine transporter; GABRA: γ-aminobutyric acid A receptor; GAP-43: growth-associated protein-43; GSK-3β: glycogen synthase kinase 3β; LTP: long-term potentiation; MARCKS: myristoylated alanine-rich C kinase substrate; NMDAR: N-methyl-D-aspartate receptor; SERT: serotonin transporter.

Extensive work on animal models of memory requiring cyclic adenosine monophosphate (cAMP) and protein kinase A (PKA) were conducted in the 1980s, emphasizing that production of new proteins for memory storage is necessary (Kandel, 2001). Around the same time, the identification of a cAMP-independent brain enzyme (Takai et al., 1977) was followed by the identification of protein kinase C (PKC), a kinase dependent on diacylgycerol and Ca++ (Takai et al., 1979). The PKC enzyme family consists of over 10 different isoforms which are involved in signal transduction from yeast to vertebrate systems (Steinberg, 2008), with its discovery and subsequent history now extensively reviewed (Kikkawa, 2019). Isoforms include classical PKCs (alpha, beta-1, beta-II, gamma) which require diacylglycerol (DAG), Ca++ and phospholipid for activation; novel PKCs (delta, episolon, eta, theta) which only require DAG and not Ca++; and atypical PKCs (iota, zeta) which require phosphatidyl serine only (Nishizuka, 1995). Immunoreactivity studies localize PKCs widely in the CNS, including in olfactory bulb, cerebral cortex (I, IV), pyriform cortex, hippocampus (CA1 stratum radiatum and oriens), amygdala (central and basolateral nuclei), and cerebellum (molecular cortex) (Saito et al., 1988). A uniquely neural tissue expressed PKC is protein kinase C gamma (PRKCG), which is exclusive to neuronal tissues compared to other PKCs widely spread in brain, heart, liver and other organs (Wetsel et al., 1992). Identified localization for PKCs are also perikarya or cell bodies of the striatum (caudate-putamen), among other brain regions (Saito et al., 1988), synaptic vesicles axon terminals (Kose et al., 1988); with PRKCG being preferentially localized in cerebellum and hippocampus (Kikkawa et al., 1988). PKC isoforms localize due to compartment and signal-organizing scaffolds using receptors for activated C kinases (RACKs), A-kinase anchoring proteins (AKAPs), heat shock proteins (HSPs) as well as annexins (AnxA1, A2, A5, and A6) (Hoque et al., 2014). The effect of PKCs in many neuronal functional systems is depicted in Figure 1, and along with the cAMP-PKA pathways, both are two important components of memory and learning systems (Lorenzetti et al., 2008).

We review below general PKC function in relation to psychiatric illness with a focus on drugs affecting PKC pathways, with emphasis on the function of PRKCG, given its almost exclusive presence in neural tissue. Based on the potential utility for pharmacotherapeutic modulations of PRKCG, this review will focus on inhibitors of PRKCG in relation to psychiatric illness. Whether PRKCG is over-expressed or under-expressed in psychiatric illness is an area of active inquiry, although the following premise can be formulated: disorders that include overactivity of mentation (obsessions in OCD, worries in anxiety states, racing thoughts in mania) may have overlapping biological correlates based on cross-disorder mechanisms. Such a biological process may result in an acceleration of mental activity. Whether this excessive mental activity is a result of enzymatic second messenger kinase overactivity remains an area for investigation. It is of interest to note that psychotropic medications that treat bipolar disorder (lithium) and anxiety states (selective serotonin reuptake inhibitors, SSRIs) appear to attenuate the activity of PKC, as discussed below, suggesting by inference that these mental states may result from an overactivity of PKC itself. In support of PKC overactivity in models of mental illness, a PRKCG KO mouse paradigm displays decreased anxiety (Bowers et al., 2000). Conversely, neurodegenerative disorders with deterioration of cognition and memory, for example, memory loss in Alzheimer’s disorder, may benefit from activators of PKC (Alkon et al., 2007).

### 1.1 Signalling mechanisms across PKC Subfamilies

Several PKC isoforms are associated with psychiatric illness, in particular, PRKCA and PRKCG have been the most studied in relation to psychopathology. In fact, multiple PKC isoforms have relevance in psychiatric conditions: PKC alpha (PRKCA) is decreased with use of lithium, resulting in modulation of GSK3-beta (Kirshenboim et al., 2004); PKC delta (PRKCD) is required for fear learning in lateral amygdala (Yu et al., 2017) and GABAergic neurons highly express PRKCD on alcohol exposure (Dilly et al., 2022); PKC epsilon (PRKCE), in particular, is decreased in an Alzheimer disease mouse model (Hongpaisan et al., 2011), mediates response to allosteric GABA-A receptors (Hodge et al., 2002), has a role in a recognition memory task (Zisopoulou et al., 2013), may mediate seizure mechanisms (Toth, 2005), mediates the neuroprotective effect of estrogen (Jung et al., 2005) and is significantly suppressed in hipoocampal CA1 in a Fragile X syndrome mouse model (Marsillo et al., 2021). Hereon the review will focus on PKC properties in general, and refer to specific PKC isoforms when appropriate, such as the predominantly brain-expressed PRKCG.

**FIGURE 2:**
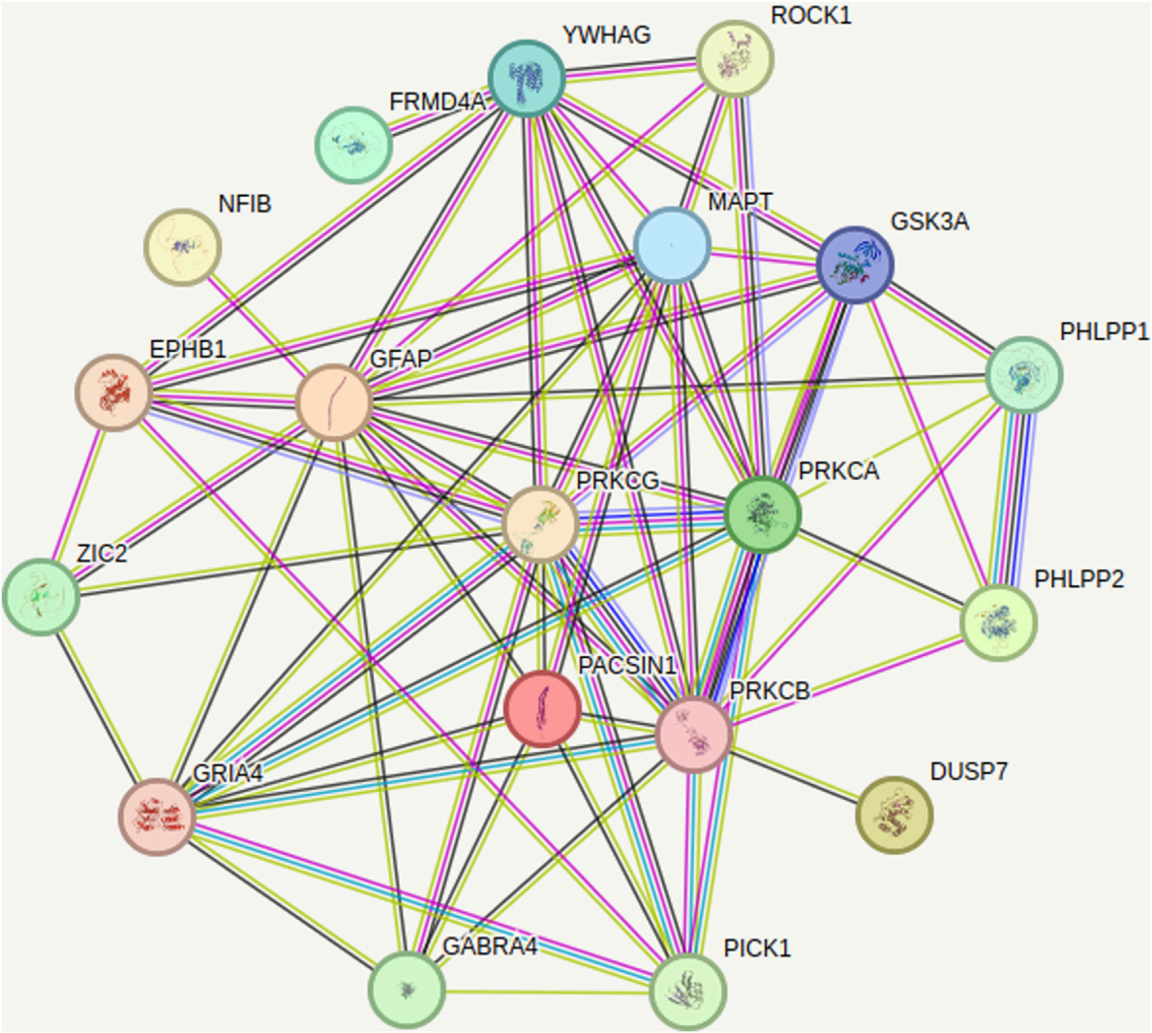
Action of PKC on Neuronal Function with Hub Protein effect of PRKCG (string-db.org). PRKGC: Protein kinase C gamma type; regulates neuronal receptors GRIA4/GLUR4 and GRIN1/NMDAR1; modulates sensitivity to opiates, pain and alcohol; mediates synaptic function and cell survival after ischemia, and inhibits gap junction activity after oxidative stress PRKCA: Protein kinase C alpha type; involved in cell proliferation and cell growth arrest by positive and negative regulation of the cell cycle PRKCB: Protein kinase C beta type; regulates B-cell receptor (BCR) signalosome, oxidative stress-induced apoptosis, androgen receptor-dependent transcription regulation, insulin signaling and endothelial cells proliferation GRIA4: Glutamate Ionotropic Receptor AMPA Type Subunit 4; receptor for L-glutamate, the main CNS excitatory neurotransmitter GABRA4: Gamma-aminobutyric acid receptor subunit alpha-4; facilitates neuronal inhibition binding GABA receptor by opening chloride channels GSK3A: Glycogen synthase kinase-3 alpha; anti-apoptotic complex; phosphorylates mTORC2 complex; facilitates amyloid precursor protein (APP) processing EPHB1: Ephrin type-B receptor; in CNS development regulates axonal guidance GFAP: Glial fibrillary acidic protein; GFAP, a class-III; cell-specific marker in CNS development for astrocytes MAPT: Microtubule-associated protein tau, promotes microtubule assembly YWHAG: 14-3-3 protein gamma; recognizes and modulates phosphoserine or phosphothreonine motifs ZIC2: Zinc Finger protein; activates the transcription of the serotonin transporter SERT; drives the expression of EPHB1 PICK1: PRKCA-binding protein; induces synaptic plasticity by regulating the trafficking and internalization of AMPA receptors; regulates acid-sensitive K+ channels ASIC1/ASIC3.

PKC activation is scaffolded by: 1) Ca++ availability due to NMDA receptor stimulation and endoplasmic reticulum Ca++ release via ryanodine receptors [RyRs] and inositol 1,4,5-trisphosphate receptors [IP3Rs] interaction, and; 2) diacylglycerol (DAG) from membrane phospholipase C acting on phosphatidylinositol-3,4,5-triphosphate (PIP3) (Fagnou and Tuchek, 1995). Functionally, translocation of PKC from the intracellular cytosol to the post-synaptic membrane is necessary for enzyme activation (Sacktor and Schwartz, 1990), a process which can be visualized through live imaging techniques (Farah and Sossin, 2011) or availability at the membrane (Hannun et al., 1986) and is readily stimulated by the use of phorbols (Weiss et al., 1989). The range of PKC activation of critical proteins is vast, with only a few examples listed: 1) activation of neurogranin (a Ca++-dependent modulator of calmodulin) (Ran et al., 2003); 2) activation of TRK receptors (Arévalo and Wu, 2006); 3) activation of cyclic AMP (cAMP) linked to CREB function (Havekes et al., 2015), adjustment of neurotransmitter release by SNAP-25 (Abrial et al., 2014); 4) arresting neurodegeneration and apoptotic signalling by GSK-3beta inhibition (Yu and Lin, 2016); 5) activation of GABA-A receptors on multiple receptor sites (Nakamura et al., 2015); and, 6) activation of glutamate transport in glial cells (Casado et al., 1991) among others. Of special significance in relation to glutamate transmission is PKC phosphorylation of the GluR1 and GluR2 components of the AMPA receptors (Xia et al., 2000; Boehm et al., 2006). When deficient at the phosphorylation sites, animal models of anxiety and depression show improvement with the atypical antidepressant tianeptine, through glutamate modulation mechanisms (McEwen et al., 2010) linked to AMPA receptor phosphorylation (Svenningsson et al., 2007). *In toto*, PKC phosphorylation functions are critical components in neuroplasticity pathways, with PKC constituting a “hub” protein for enzymatic systems impacting neuronal function (Figure 2) (Zaidel-Bar et al., 2007).

After the discovery of PKC, animal models used for the study of memory were applied to Ca++-dependent pkc. A central role for pkc in neuroplasticity mechanism was recognized due to its protagonism in the maintenance of Hebbian neuroplasticity based on the concepts of experience-dependent learning based on long-term potentiation (LTP) (Nicoll, 2017). In their landmark 1973 paper, Lomo and Bliss recorded neuronal connection changes that were long-lasting and increased the strength of synaptic transmission, establishing the concept of LTP (Bliss and Lomo, 1973) Most of the learning and memory research had favored PKA in the 1980s (Kandel et al., 1986), but in the 1990s pkc-mediated associative (conditioned) learning models were studied in both the marine sea snail *Aplysia* (Kruger et al., 1991) and the sea slug *Hermissenda* (Matzel et al., 1990; McPhie et al., 1993). In rats, LTP is induced by tetanic stimulation of the hippocampus, which in turn generates translocated pkc activity two-fold at the cell membrane, a required context for LTP consummation (Akers et al., 1986). Likewise in rat hippocampus, the pkc activator phorbol ester induces maintenance of LTP (Lovinger and Routtenberg, 1988), with pkc activity being required for multiple steps in memory consolidation (Sossin et al., 1994), while spatial learning effectively causes a redistribution of pkc (Olds et al., 1990). When rabbit hippocampal cells are examined after a nictitating membrane conditioned response, up to three-fold activation of pkc is noted in synaptosomes of hippocampal CA1-3 regions (Sunayashiki-Kusuzaki et al., 1993), or to the stratum oriens of CA3 (Scharenberg et al., 1991), suggesting that pkc activity underlies associative learning processes. Interestingly, avoidance learning-an anxiety response-appears to depend on pkc activity as well, as it can be blocked by immediate polymixin B, a pkc and calcium/calmodulin-dependent kinase II inhibitor, applied to rat amygdala (Walker and Gold, 1994). In summary, pkc activity is key to LTP maintenance which underlie learning and memory functions.

### 1.2 Potential role of protein Kinase C in psychiatric illness

The massive research in learning and memory processes in the 1990s did not clearly differentiate between the different isoforms of PKC, although, initial experiments identified prkcg as the main isoform associated with learning and memory as it is exclusively found in central and peripheral neurons (Wetsel et al., 1992). The notion that pkc activity is highly relevant to learning and memory function stems from experiments in the 1980s. In 1986, Routtenberg et al. proposed that a the phospholipid/Ca++ dependent “brain pkc” participated in the expression of neuroplasticity and demonstrated this effect using the phorbol acetate TPA to enhance the action of pkc to maintain LTP in rat hipoccampal neurons (Routtenberg et al., 1986), in effect, producing a “a long-lasting potentiation of synaptic activation” (Colley et al., 1989). By 1997, the role of pkc in animal models of LTP for learning and memory was summarized by Nogues et al. (1997) (Noguès, 1997). Several key properties of pkc are noted: 1) pharmacologically, pkc is promoted by the tumor-enhancing and synaptic plasticity-promoting phorbol esters; 2) this activation is associated with the maintenance stage of LTP (Linden and Routtenberg, 1989); 3) LTP persistence, in turn, can be blocked by pkc inhibitors such as polymixin B, depending on the timing of the drug application (Lovinger et al., 1987); and, 4) in animal studies, pkc has a critical role in learning paradigms, for example, imprinted learning in newborn chicks via phosphorylation of the neuron growth-associated protein GAP43 (Sheu et al., 1993). Given this body of research, further exploration into PKC and its isoforms has been undertaken in relation to psychiatric illness.

### 1.3 PRKCG: a more specific candidate for exploration in psychiatric illness

Multiple experiment starting in the early 1990s found specificity for the PRKCG isoform in neuronal function. In 1992, in a series of amygdala-kindling experiments, Belduis et al. demonstrated that spatial discrimination tasks increased CA1 and dentate gyrus prkcg (but not prkca, prkcb-I, nor prkcb-II); while also documenting the inverse, with prkcg decreasing function leading to loss of ability to complete tasks due to seizures (Beldhuis et al., 1992). Later, van der Zee et al. showed that spatial and associative learning in rat, mice and rabbit are coupled to pkc immunoreactivity (ir), selective for the gamma-isoform (prkcg). The level of prkcg ir is also associated with the degree of learning ability (poor learners vs good learners) and during learning, decreased pkc beta-II activity in astroglia is accompanied by increased ir prkcg activity in dendritic spines; the authors postulate that a facilitating ir-detected conformational change in prkcg may have occurred (Van der Zee et al., 1997). With pkc beta (prkcb) playing a role pre-synaptically, prkcg was identified as the isoform in the post-synapse singled out for its role in triggering the second messenger cascade critical for LTP (Colley and Routtenberg, 1993). The subcellular distribution of prkcg soon became an area of interest, and the Golgi apparatus was identified as the compartment harboring high concentrations of DAG and prkcg (Cardell et al., 1998). Being exclusively expressed in central and peripheral neurons, prkcg activity specifically sustains LTP expression by engaging pre- and post-synaptic substrates, as well as metabotropic glutamate (mGluR) pathways (Ramakers et al., 1997). The LTP-sustaining property of prkcg marked it as a key molecule in memory generation and maintenance, with over 100 substrates of prkcg identified by 1999 (Riedel, 1997).

In summary, the breadth of PKC modulators may open windows into new therapeutic approaches using repurposed or newly developed pharmaceutical products. Based on the role of PKCs, and PRKCG in particular, it is critical to revisit the potential use of drugs that modulate PKC activity to explore psychiatric treatments, given PKC’s role in memory and learning via LTP, and its wide-ranging span of biological activity in the CNS. In this exploratory review, the role of PKC in neuropsychiatric conditions is examined from neurobiological, pre-clinical and clinical perspectives. An overarching theme in this exploration is that therapeutic agents that downregulate PKC, and particularly PRKCG, appear to have benefit in reducing neuropsychiatric symptom expression. This is seen in the use of SSRIs and antidepressants for anxiety and depressive states, lithium and tamoxifen in bipolarity, where pre-clinical studies show downregulation of PKC activity and clinical data show improved outcomes.

## 2 PKC inhibitors

### 2.1 Antidepressants

#### 2.1.1 Fluoxetine

Fluoxetine, like other antidepressants, is known to positively affect neurogenesis and neuroplasticity with chronic use (Higuchi and Arakawa, 2023). Early studies demonstrated that antidepressants (fluoxetine, reboxetine or tranylcipromine) generate division of neurons in the dentate gyrus and hilus of the hippocampus. Using bromodeoxyuridine (BrDU) tagging for cell division, hippocampal cell BrDU tagging increased by 20%–40% with antidepressants, and 50% with electroconvulsive therapy (Malberg et al., 2000). The observation that chronic administration of fluoxetine increases neuronal proliferation and protects immature neurons in the hippocampus is replicated in several studies (David et al., 2010). In these studies, specific to fluoxetine, the following are observed: 1) specificity for type of progenitor cell affected by fluoxetine (Encinas et al., 2006); 2) causal connections made between neurogenesis induction and fluoxetine-driven behavioral changes in mice (Santarelli et al., 2003), and; 3) increased dendritic arborization from chronic fluoxetine use (Wang et al., 2008). More recently fluoxetine has been found to generate induced-plasticity (“iPlasticity”), as demonstrated by fluoxetine-driven a) increased plasticity in the visual cortex after light deprivation; b) fear network erasure; c) extinguished aggression caused by social isolation, and; 4) facilitation of spatial reversal memory in rodent models (Umemori et al., 2018). Hence, chronic, but not acute, use of fluoxetine is linked to neuroplasticity induction.

The effect of chronic fluoxetine on pkc expression was expressly examined by Rausch et al. (2002). Measuring kinase mRNA expression with a gene chip in rats treated with a 3-day or 21-day courses of fluoxetine, specific pkc isoforms prkcg and prkcd were found to be increased after 3 days and decreased after 21 days of fluoxetine administration (Rausch et al., 2002). Earlier studies had noted that decreased pkc expression facilitates neuronal proliferation. In 1995, Mann et al. investigated the expression of pkc after chronic 21-day administration of fluoxetine in rats, finding a pkc decrease of 18% in hippocampus CA1 and a 28% decrease in the paraventricular thalamic nucleus; notably a single administration of fluoxetine had little effect (Mann et al., 1995). Thus, a pattern of decreased prkcg follows chronic use of fluoxetine in these two prior experiments. The connection with increased arborization and decreased prkcg was examined by Keeler et al. (2015). In the experiment baseline, gamma-protocadherin adhesion molecules were shown to regulate dendrite arborization by inhibition of focal adhesion kinase (fak), which modulates gamma-protocadherin. However, fak is activated by prkcg phosphorylation of a C-terminal fak serine, with fak-activation disrupting the neuroplasticity-promoting gamma-protocadherin. The effect of prkcg phosphorylation is to reduce dendrite arborization by impairing the ability of gamma-protocadherin to promote arborization; in this context, reduced prkcg promotes neuroplasticity (Keeler et al., 2015).

It has been shown that fluoxetine suppresses pre-synaptic glutamate release independent of exocytosis. This neuroprotective suppression of glutamate release is mediated by Ca++ channels (P/Q type), a step which is modulated by pkc and not pka. When pkc is activated the fluoxetine glutamate-reducing neuroprotective effect is abolished (Wang et al., 2003). In a follow-up experiment Lazarevic et al. (2019) found that pkc modulated both glutamate and GABA release (Lazarevic et al., 2019). In summary, fluoxetine appears to act therapeutically, at least in part, through the promotion of neuroplasticity, with one of the potential mechanisms including reduction of PRKCG activity.

#### 2.1.2 Tricyclics

Tricyclics have been used in the treatment of depression, anxiety and other conditions since 1958, when Roland Kuhn informed the field of the use of imipramine for depressive disorders, naming this drug class ‘thymoleptics’ (Kuhn, 1958). After the introduction of various tricyclics and selective serotonin re-uptake inhibitors (SSRIs), two competing ideas on the action of antidepressants became current, given the observed effectiveness of antidepressants only on chronic use: receptor desensitization and the modulation of second messenger systems (Feighner, 1999). Starting with the observation that 5-HT receptors stimulate hydrolysis of phosphoinositides to produce IP3 and DAG second messenger molecules (Berridge, 1987), Bush-Sanders et al. showed that 28-day use of sertraline (but not 14 days) decreased production of IP3 and DAG, the activators of the pkc cascade, without a change int the affinity or in the density of the 5HT-2 binding site. A similar effect was observed with amitriptyline; in both instances, phospoinositide hydrolysis was unchanged if norepinephrine was used to stimulate hydrolysis-suggesting the effect is specific to 5-HT (Sanders-Bush et al., 1989). Exploring the effect of pkc on the cAMP pathway, Nalepa et al. observed that noradrenaline or isoproterenol (epinephrine analog) induces cAMP accumulation, which is further driven by PKC activity. On administration of imipramine, the pkc potentiation of the noradrenaline-cAMP response is attenuated, suggesting that imipramine can directly inhibit pkc activity (Nalepa and Vetulani, 1991). Based on the notion that phosphoinositide hydrolysis, pkc activity and GAP43 (substrate of PKC for neuronal growth) are involved in supporting neurite outgrowth, and the observation that neurite growth is inhibited by amitriptyline (Farbman et al., 1988), Wong et al. examined the effect of amitriptyline on phospholipase C (PLC), pkc and cAMP. In cerebral explant cultures, DAG analogues (diolein, dilinolein) had a significant stimulatory effect on neurite outgrowths. With amitriptyline, neurite outgrowth was diminished through inhibition of PLC activity through a guanine-nucleotide dependent mechanism; additionally, when several substrates were tested, those with pkc phosphorylation were the most inhibited by amitriptyline use (Wong et al., 1993). Using rat brain homogenate for enzyme extraction, Morishita et al. (1997) tested the effect of the tricyclic desipramine on pkc activity. Increasing concentrations of desipramine at 0.1, 1 and 10 mmol/L significantly decreased pkc activity (Morishita and Watanabe, 1997)**.** In 2002, Dwivedi et al. pursued the notion that antidepressants, rather than involving specific neurotransmitter and receptor mechanisms, acted by modulating second messenger transduction systems by affecting the PKC pathway via membrane PLC changes. Specifically, targeting the antidepressant-- > receptor-- > G(o)/G(q) GTP binding protein-- > phospholipase (PLC; DAG, IP3, Ca++)-- > PKC pathway, acute and chronic use of desipramine or fluoxetine in rat hippocampus and cortex preparations were assayed. Acute use of either desipramine or fluoxetine did not alter IP3 or PLC activity in membrane or cytosol, but 21-day chronic use of either antidepressant produced a significant decrease in IP3-PLC activity in both membrane and cytosol, specific to PLC-1beta, suggesting that chronic antidepressant use depressed the PKC pathway (Dwivedi et al., 2002). In summary, a number of experiments with tricyclics in relation to pkc activity demonstrate a robust decrease of the enzyme activity with chronic tricyclic use.

More contemporaneous work on the effect of tricyclics on pkc systems identify specific affected isoforms. Amytriptyline downregulation of prkcg was examined by Tai et al. (2007) using a model of upregulation of pka and pkc in the spinal cord dorsal horn by long-term morphine infusion. Additionaly amitriptyline inhibits the increase in expression of phospho-pka, prkca, prkcb-II, and prkcg, accompanying relief of chronic pain. The mechanism for this effect involved downstream decrease of excitatory amino acid (EAA) by more efficient resorption of glutamate and other EAAs via glutamate transporters in microglia after amytripline use (Tai et al., 2007). Models of stress in mice have used the forced swim test (FST) (Commons et al., 2017) to examine the effect of stress on the pkc system, and its response to antidepressants. Using the FST in mice, prkce phosphorylation was increased on stress exposure, modeling the coping with stress response, however, in this experiment, the only isoform tested was prkce (Galeotti and Ghelardini, 2012). The same research group then investigated the inositol triphosphate (IP3)-phospholipase (PLC)-PKC cascade in other isoforms. Blocking the PKC pathway with calphostin C, less FST immobility time was elicited, a known anti-depressant effect. Using antisense oligonucleotides to block protein expression, a selective knockdown of PLC-beta1 and prkcg were identified in the causal chain of the FST effect, suggesting that the tricyclic antidepressant effect depends specifically on decreasing PCL-prkcg activity (Galeotti and Ghelardini, 2011).

In summary, tricyclics are similar to fluoxetine in attenuating the activity of pkc species, and in several instances of prkcg specifically. This attenuation lends support to the notion that the second messenger system is in a state of over-activity in mood and anxiety disorders, which can then be ameliorated by chronic (>21 days) use of antidepressants through inhibition of overactive second messenger system components, in the end, allowing for enhanced neuroplasticity mechanisms to take effect.

#### 2.1.3 St. John’s Wort. St. John’s wort (*Hypericum perforatum)* has been used for over 2,000 years for medicinal purposes

One of the main active ingredients of this herbaceous plant is hypericin, and since its isolation in 1957, extensive research has been conducted on hypericin’s biochemical properties, biosynthesis and delivery system capabilities (Wu et al., 2023). In neurons, the effect of hypericin has been examined in rat cerebral cortex, showing inhibition of glutamate release in a dose-dependent manner by diminishing pre-synaptic vesicular exocytosis. A mechanism involving N- and P/Q voltage-dependent Ca++ channels and the MAPK pathway was identified, acting through inhibition of glutamate release (Chang and Wang, 2010). In relation to pkc, a strong inhibitory effect of hypericin has been extensively documented. In 1989, Takahashi et al. reported that a pkc inhibitor, calphostin C, had a similar structure to hypericin, with both also demonstrating anti-retroviral activity. They further showed that hypericin inhibited pkc but not pka, by attaching to pkc’s uniquely configured regulatory domain (Takahashi et al., 1989). Other reports also highlight the breadth of hypericin’s inhibition of protein kinases in growth factor signalling pathways, including the inhibition of protein tyrosine kinase (ptk) and pkc in a photosensitive manner (Agostinis et al., 1995). Interestingly, other reports confirm the light sensitivity of pkc activity in this context (Bruns et al., 1991), with hypericin inhibiting pkc activity in a light- and concentration-dependent manner (Utsumi et al., 1995). More specifically for prkcg, using male Swiss albino mice, Galeotti et al. (2013) report that in mice treated with nociceptive stimulators, periaqueductal grey and thalamus have a higher expression of prkcg and prkce pain-modulating isoforms. On administration of hypericin there was a complete blocking of the increase of prkcg phosphorylation induced with the nociceptive stimulators (Galeotti et al., 2010). For mild-moderate depression meta-analyses show utility of St. John’s wort across more than 3,800 participants in 27 clinical trials (Ng et al., 2017), supporting the notion that at least in part, inhibition of PKC plays a role in the effect of St. John’s wort in anxiety and depressive states.

### 2.2 Mood-stabilizers

#### 2.2.1 Lithium

The role of PKC in the pathophysiology of bipolar disorder was suggested in 1994 by Manji and colleagues after observing that chronic lithium use has PKC-dependent transcriptional and posttranscriptional effects involving long-term neuroplasticity and cellular response. Relevant to the mechanism of PKC downregulation by antidepressants and valproate (see below), acute lithium use mimics the effect of phorbol esters with resultant activation of PKC (energizing immediate early genes such as c-fos), while chronic lithium use attenuates the phorbol ester action on PKC, down-regulating the enzyme (Manji and Lenox, 1994). In a series of trials, this same research group found that use of lithium reduced PKC in rat frontal cortex and hippocampus, suggesting that “PKC inhibition may have antimanic efficacy”. The main isoforms studied in these PKC studies were prkca and prkce (primarily expressed in cardiac tissue), with less emphasis on prkcg (Manji et al., 1999). By 2008, the proposition that anti-manic drugs attenuated kinases for their therapeutic effect was put forth, including key kinases GSK3 (inhibited by lithium, valproate and ECT) and PKC (inhibited by chronic lithium, valproate and ECT) (Catapano and Manji, 2008). More recently, the pathophysiological pathways in the genesis of bipolar disorder have expanded to non-canonical pathways, while continuing to include PKC activation as one of the putative mechanisms in the etiology of mania (Machado-Vieira et al., 2023). In a proof-of-principle autopsy study, protein and mRNA expression of PKC isoforms in prefrontal, cingulate and temporal cortex were examined in deceased participants with schizophrenia, bipolar disorder and healthy controls. A significant decrease in PKC activity in the cytosol and membrane fractions of prefrontal and temporal cortex was present in participants with bipolar disorder, but not schizophrenia, affecting all classic PKC isoforms (PRKCA, PRKB-I, PRKB-II, PRKCG) (Pandey et al., 2020). A number of studies in the 1990s confirmed dysregulation of PKC isoforms in symptomatic bipolar disorder, with the most consistent finding being an increase in PKC activity associated with manic symptoms. It is important to note that in these studies, specific isoforms are often not reported, or they are reported to include classic PRKCA and PRKCE, possibly due to their availability in non-neuronal cell lines (platelets) (Wang et al., 1999). These studies led Hahn et al. (1999) to hypothesize that “changes in PKC may be an illness-specific marker” for bipolar disorder, with enhanced PKC activity during mania being suppressed following mood-stabilizer treatment as manic symptoms improve (Hahn and Friedman, 1999). Interestingly, the mechanism by which lithium may attenuate PKC activity may reside in the prevention of translocation from the cytosol to the membrane where DAG and Ca++ are available to activate PKC. This translocation is facilitated by K+ and phorbol esters, and the availability of Ca++ partially abolishes the lithium effect (Wang et al., 2001). A more recent intriguing approach to the potential action of lithium in attenuating PKC over-activity has been proposed, suggesting that monovalent Li + can displace the bulkier divalent Ca++ in order to inhibit PKC downstream activity. Testing this approach using modeling computational techniques, either PRKCA and PRKCG (but not PRKCB isoforms) are vulnerable to the substitution of divalent Ca++ by monovalent Li + via the C2 domain of cytosolic unbound-to-membrane PKC, suggesting that this could be a mechanism whereby Li + impedes PKC activation (Grauffel et al., 2021). Finally, cross-talk appears to occur between the PKC cascade and cAMP amplification of the second messenger system. Increased pkc activity in an *in vivo* microdialysis experiment results in higher cAMP dialysate levels in hippocampus and cortex. Use of lithium then decreases pkc activity along with an attenuation of levels of cAMP, potentially normalizing the two classically established second messenger pathways known for associative learning (Chen et al., 2000). In summary, there is evidence for PKC overactivity in bipolar disorder, which is attenuated with anti-manic agents, including lithium, as symptoms improve. At least in part, these studies suggest that anxiety, depressive and manic states may share PKC over-activity as a common biologic marker or etiologic component, with these studies focusing mostly on prkca and prkce, with some reference to prkcg.

The link between bipolar disorder and anxiety disorders also yields clues to their possible common original pathophysiology. Multiple studies of the clinical precursors of bipolar disorder identify anxiety disorders as a common early clinical manifestation in prospective (Egeland et al., 2012), monitoring of offspring of bipolar parents (Duffy et al., 2013) and psychopathology community sample (Johnson et al., 2000) studies. Yet, most studies also support the confluence of anxiety disorders (separation, panic, generalized) and disruptive disorders (attention-deficit hyperactivity disorder, conduct disorder) as the required precursors for a later life diagnosis of bipolar illness (Faedda et al., 2015). Unopposed antidepressant therapy is known to lead to manic switch in a modest proportion of bipolar disorder patients (Gorwood et al., 2016), but this does not occur in the context of a mood-stabilizer or neuroleptic use (Bhowmik et al., 2014), highlighting the complex effects of antidepressants in specific vulnerable populations, beyond its effect on PKC attenuation. Thus, the use of antidepressants in bipolar patients has been a vital component of long-term stability (Post et al., 2003), with attention being paid to the subgroup of patients susceptible to antidepressant-induced manic switch. In one study, these patients tended to be more male and have a family history of bipolar disorder, experiencing manic switch 2–3 weeks after antidepressant treatment for depression (Wada et al., 2006). As both antidepressants and mood-stabilizers can attenuate PKC overactivity to treat depression and mania, respectively, the interplay of other factors will be important to consider in future studies of bipolar disorder in relation to PKC activity.

#### 2.2.2 Valproate

Valproate is a short chain fatty acid, derivative of valeric acid found in valerian, a medicinal herb used since ancient times for medicinal purposes (Upton, 2001). Valproate itself has multiple actions including modifying GABA levels by inhibiting 4-aminobutyrate aminotransferase (ABAT, GABA transaminase) and aldehyde dehydrogenase five family member A1 (ALDH5A1) which degrades GABA, thereby increasing inhibitory GABA availability. Valproate additionally blocks voltage-gated Na+, K+ and Ca++ channels (controlling propagation of electrical signals) and inhibits histone deacetylases (increasing signaling of apoptosis) (Ghodke-Puranik et al., 2013)**.** The effect of valproate on pkc has been examined in conjunction with the observation that mood-stabilizers have an attenuating effect on the pkc system. In a *Caenorhabditis elegans* model, valproate inhibited behaviors associated with inositol triphosphate (IP3) and DAG, which was countered by administration of phorbol ester, a pkc activator. The valproate effect was traced to inhibition of PLC which forms DAG and IP3 and would hamper downstream pkc activity (Tokuoka et al., 2008). Using immortalized hippocampal cells, Watterson et al. sought to test the effect of chronic 14-day valproate on two markers of neuroplasticity, myristoylated alanine-rich C kinase substrate (MARCKS) and GAP-43, both known PKC substrates. While valproate predictably reduced the expression of the pkc substrate MARCKS, it enhanced the expression of gap-43 concurrent with increased neurite outgrowth. Interestingly, early use of valproate (up to 3 days) increased pkc membrane activity, but later (14 days) significantly reduced pkc activity, an effect similar to that observed with fluoxetine and lithium. While cell number was not increased, neurite outgrowth increased with chronic valproate or use of a pkc inhibitor (LY333531), reaching peak neurite outgrowth when both were used (Watterson et al., 2002). These observations replicate the findings of Keeler et al. (2015) wherein pkc activation of fak decreases neurite outgrowth, while pkc attenuation has strong neuroplasticity effects via fak-gamma-protocadherin interaction. When valproate-treated adult rats are examined for PKC phosphorylation of substrates NMDAR1 and MARCKS, there is a significant decrease in immunoblotting tests of phosphorylation by pkc, indicating a downregulation of hippocampal pkc activity due to valproate (Watson et al., 1998). Specific to prkcg, valproate can inhibit the activation of prkcg by decreasing the membrane load of prkcg, with resultant adverse effects on spatial memory, results similar to those produced by carbamazepine and phenobarbital (Wu and Wang, 2002).

Valproate has varying effects depending on the stage of brain development, as it can be toxic in embryogenesis (Chomiak et al., 2013). Early fetal exposure to valproate is teratogenic in humans (Laegreid et al., 1993) and prenatal valproate in rodents has been used to model autism in rats (Ingram et al., 2000), mice (Yochum et al., 2008) and zebrafish (Li et al., 2024). In summary, the action of valproate in neural systems is complex, with one aspect including downregulation of PKC signaling, which overlaps with symptom amelioration of manic states in animal models, suggesting that modulation of PKC overactivity is a mechanism that is favored by valproate.

#### 2.2.3 Tamoxifen

While first considered to be a potential cancer therapy agent due to its ability to inhibit PKC activity (O’Brian et al., 1985), tamoxifen later emerged as a drug candidate for mood stabilization given its PKC-inhibiting properties. In 1985, O’Brian et al. (1985) reported that tamoxifen, the nonsteroidal anti-estrogen used to treat breast cancer, inhibited PKC, which was then known to bind with high affinity to tumor-promoting phorbol esters (O’Brian et al., 1985). This experiment was motivated by the observation that tamoxifen inhibited bovine brain cAMP phosphodisterase. With a mechanism related to Ca++ influx, tamoxifen appears to operate through Ca++-sensitive PKCs, including prkca, prkcbi, prkcbii and prkcg (Radin and Patel, 2016). Later experiments have demonstrated that PKC can be translocated to the membrane with tamoxifen, resulting in feedback downregulation of PKC activity (Gundimeda et al., 1996). Its use in cancer to date benefits from its PKC inhibiting actives as well as its selective estrogen receptor modulator property in hormone receptor-positive breast cancer (Nelson et al., 2019), while its use as a mood-stabilizer can is under development. Tamoxifen has an active metabolite, 4-OH-tamoxifen which easily crosses the blood-brain barrier (Lien et al., 1991), setting up its central action, while competitively inhibiting binding of estradiol to estrogen receptor alpha (ER-alpha) and estrogen receptor beta (ER-beta) (Kuhl, 2005). Interestingly, tamoxifen acting mostly as an anti-estrogen compound has potential for causing cognitive side effects (Novick et al., 2020), given the neuroprotective property of central estrogen, but the true effects of tamoxifen as an estrogen antagonist or agonist appear to depend on the background concentration of endogenous ligand (partial agonist at low estrogen concentration, for example,) (Paige et al., 1999). Animal models of mania have consistently supported the use of tamoxifen as an anti-manic agent, using the amphetamine-induced mania hyperactivity model (137,138**;** 139; 140), while additionally reporting additional beneficial effects on cytotoxic oxidative stress (Steckert et al., 2012).

The clinical use of tamoxifen in at least one case series and six randomized clinical trials (RCT), albeit modest in size, continue to suggest a potential utility for treatment of bipolar mania. A single blind case series of tamoxifen in seven patients with bipolar disorder (Bebchuk et al., 2000) was followed by small RCTs which show significant improvement over placebo in 13 women with mania (Kulkarni et al., 2006), 16 patients with mania/mixed states (Zarate et al., 2007) and 21 adults with manic/mixed states (Yildiz et al., 2008). An adjunct-to-lithium trial also shows positive results with tamoxifen 80 mg + lithium 1.0–1.2 mEq/L being superior to placebo adjunct (Amrollahi et al., 2011). Additional studies of tamoxifen in bipolar illness include an RCT enrolling 51 adult females with mania treated with lithium, valproate or carbamazepine, using tamoxifen, medroxiprogesterone acetate (MPA) or placebo as adjuncts. Both tamoxifen 40 mg and MPA were beneficial in ameliorating manic symptoms compared to placebo, especially in the first week of treatment (Kulkarni et al., 2014). In an RCT of 44 youth assigned to lithium only or lithium + tamoxifen 20–40 mg, the augmentation with tamoxifen group had significantly more 50% reduction proportion in the active treatment augmentation group for mania ratings compared to placebo augmentation (Fallah et al., 2016). By 2016, a review of tamoxifen trials in bipolar disorder concluded that tamoxifen was effective in the short-term treatment of mania, with results similar to lithium or valproate, with about 50% response rate across all three drugs. No serious side effects were reported in these short-term trials, however, it should be recognized that long-term tamoxifen use is associated with endometrial cancer, uterine sarcoma and even depression (Talaei et al., 2016), due to the observation that in cancer cohorts mood symptoms worsen with long-term tamoxifen use in those predisposed to depression (Carmassi et al., 2021). In summary, tamoxifen is a central PKC inhibitor with anti-manic properties, however, to date the RCTs using tamoxifen as single or augmenting agent are relatively small in size and larger clinical trials are needed to support definitive efficacy (Kishi et al., 2022).

### 2.3 Glutamate modulators

#### 2.3.1 Riluzole

Glutamate excess can induce CNS neurotoxicity, leading to interest in developing a class of drugs which modulate glutamate activity directly or indirectly (Grados et al., 2015). Among these, riluzole (2-amino-6-trifluoromethoxy benzothiazole) is a neuroprotective drug approved for use in amyotrophic lateral sclerosis (Miller et al., 2002), which appears to act through decreasing glutamate release by stabilizing inactive Na + voltage-gated channels, shifting voltage dependence of the channel in the hyperpolarizing direction (Herbert et al., 1990). Other glutamate-attenuating neuroprotective mechanisms have been proposed for riluzole (Kim et al., 2007; Fumagalli et al., 2008; Gourley et al., 2012), although GABA potentiation may also occur (He et al., 2002). Its use in psychiatric disorders is still an emerging area of research (Pittenger et al., 2008; de Boer et al., 2019), and clinical applications have been explored for mood symptoms in autism spectrum disorder (Ghaleiha et al., 2013), treatment-resistant schizophrenia (Pillinger et al., 2019), treatment-resistant obsessive-compulsive disorder (Grant et al., 2007), and methamphetamine dependency (Farahzadi et al., 2019) among others. Given the potential for neuronal oxidative stress damage in ALS and the leading role of increased PKC in ALS, Noh et al. (2000) explored whether riluzole inhibits PKC. Using a neuronal cortex culture, riluzole blocked the stimulation of pkc by phorbol esters reducing oxidative neuronal death by binding the catalytic domain of pkc (Noh et al., 2000). Using another approach to understand its neuroprotective effect, riluzole decreased glutamatergic transmission via inhibition of pkc, based on pkc regulation of presynaptic NMDA receptors which facilitate glutamate release. As Lamanauskas et al. (2008) suggest, “controlling NMDA receptor function and, thus, excitatory transmitter release via modulation of pkc, suggests a novel potential target to counter glutamate excitotoxicity” (Lamanauskas and Nistri, 2008). In a more recent experiment, Lazarevic et al. (2018) sought to determine the effect of riluzole by measuring the impact of riluzole on presynaptic activity, synaptic vesicle recycling and glutamate release. The data suggest that riluzole selectively lowers the readily releasable pool through a pkc-mediated mechanism involving Munc18-1. Munc18-1 promotes syntaxin stability and controls the spatial assembly of core complexes for SNARE-dependent fusion, making pkc phosphorylation of Munc18-1 the crucial step in neurotransmitter synaptic vesicle load (Lazarevic et al., 2018). The sum of the data in relation to riluzole suggests that riluzole’s main mechanism may not wholly depend on PKC downregulation, a known property of the drug, yet PKC downregulation may provide added benefit to other riluzole effects for an overall ultimate neuroprotective effect.

### 2.4 Antioxidants

#### 2.4.1 N-Acetylcysteine (NAC)

NAC is a synthetic acetylated derivate of the endogenous amino acid L-cysteine, a precursor of glutathione, a powerful free radical scavenger of reactive oxygen species (Ziment, 1988). Glutathione (L-gamma-glutamate-cysteine-glycine) is the most prevalent intracellular thiol (Meister and Anderson, 1983), and in the cytosol, glutathione-dependent oxidoreductases regulate redox signal transduction as well as protect protein thiols from irreversible oxidation (Chai and Mieyal, 2023). Beyond its direct effect in ameliorating cellular oxidative stress, NAC has a role in the modulation of glutamate homeostasis. A mouse KO model of the glutamate transporter slc1a1 showed a decreased load of intracellular glutathione as well as premature brain aging; NAC treatment reversed these changes (Cao et al., 2012). More specifically, NAC inhibits the release of excitatory glutamate by activation of pre-synaptic mGluR2 (Kalivas, 2009). This last action appears to involve Sxc- (cysteine/glutamate antiporter, or system xc –), a system present only in vertebrate brains. NAC directly activates Sxc-, which signals pre-synaptic mGlu2 to modulate the glutamate excitatory load. When Sxc-is abolished in animal models, behaviors such as approaching foods on cue even when punished, perseverative errors and cocaine-primed drug seeking are increasingly present (Hess et al., 2023).

The action of NAC on prkcg activity has been explored in adult male rats in a neuropathic pain model. The use of a neuropathic pain model involving prkcg has been current for many years (Malmberg et al., 1997), with Li et al. (2016) using a chronic constrictive injury (CCI) model on the sciatic nerve in rats to show NAC inhibition of matrix metalloproteinase (MMP). In this model, prkcg is known to play a facilitating role, since PRKCG sensitizes neurons to pain via NMDA receptors. The authors observed that use of NAC had a dramatic effect in decreasing prkcg phosphorylation (Li et al., 2016). A similar outcome is reported by Liu et al. (2017) with NAC suppression of the phosphorylation of prkcg resulting in lower allodynia load (Liu et al., 2017). In summary, there is limited but extant evidence for a decrease of prkcg activity on administration of NAC in pre-clinical models.

#### 2.4.2 Myricitrin

Myricitrin is one of 5,000 currently known flavonoids, and is a derivative of trees and shrubs, among which are the noted Morella and Myrica species (Myricaceae) (Rosa et al., 2020). As a botanical flavone with strong antioxidant properties and abundant in multiple plants (Zhang et al., 2020), the extraction of myricitrin has been streamlined, with methods of high-level production under exploration (Geng et al., 2023). Recently, a review of its potential use in anxiety and depression highlights its potent anti-PKC properties (Ko et al., 2020). An examination of myricitrin’s anxiolytic properties was carried out using the elevated plus maze (EPM) in mice, showing that myricitrin effectively increased animal time on the EPM, an anxiolytic effect, without causing sedation or the myorelaxation seen with diazepam (Fernandez et al., 2009). In a model of psychosis using apomorphine-induced stereotypy and the climbing/paw test in Swiss albino mice, myricitrin decreased the psychosis-probing behaviors without affecting locomotor behaviors or producing catalepsy through a pkc inhibition mechanism (Pereira et al., 2011). The PKC inhibition properties of myrycitrin have been widely documented (Gamet-Payrastre et al., 1999), and potentially affect all PKC pathways without specificity for isoforms (Meotti et al., 2006). In summary, myricitrin is a promising anxiolytic, and possibly anti-psychotic, in part based on its PKC inhibition properties.

### 2.5 Alkaloids

#### 2.5.1 Chelerythrine

Microbial alkaloids such as stauroporine are known PKC antagonists, but in general they are non-selective for PKC, while chelerythrine inhibits PKC but not PKA, CamK, or tyrosine protein kinase (TPK). Chelerythrine inhibits PKC in a concentration-dependent manner by binding to the catalytic ATP or protein-binding site, and not the regulatory region where DAG or phorbol ester bind (Herbert et al., 1990). On the other hand, chelerythrine has other effects, for example, it can affect multiple ion channels in cardiac tissue in a pkc-independent manner (Son et al., 2011), but also anti-inflammatory effects via a pkc mechanism, with pkc acting as an upstream regulator of the pro-inflammatory PKC/NF-kappaB pathway (Zeng et al., 2015). The sleep deprivation rat model of mania induces hyper-locomotion, increased sleep latency, and reduced hippocampal cell proliferation, with markers of pkc overactivity such as excess phosphorylated hippocampal and prefrontal synaptosomal-associated protein 25 (SNAP-25). In this context, chelerythrine improves the manic-like manifestations and reverses the decreased cell proliferation in the hippocampus (Abrial et al., 2014). While the effect of acute chelerythrine is to decrease mania in the sleep deprivation rat model, chronic 14-day exposure caused depressive-like behaviors in one study (Abrial et al., 2013). However, using the FST model of depression in Swiss albino mice, chelerythrine and other pkc inhibitors (calphostin C, neomycin) produce an antidepressant effect (reduced FST immobility time). In this experiment, pkc isoforms were examined, and the effect was diminished when prcg specifically was targeted, suggesting prkcg supported the antidepressant effect of the pkc inhibitors, with the authors concluding that “an over-stimulation of PRKCG might be present in depressed patients and that the inactivation of PRKCG may represent a new target for the antidepressant therapy” (Galeotti and Ghelardini, 2011). In summary, chelerythrine is a relatively selective PKC inhibitor with anti-manic properties in animal models as well as an antidepressant effect specific to prkcg. No human trials using chelerythrine are known to date.

### 2.6 NMDA antagonists

#### 2.6.1 Ketamine

Ketamine is a dissociative anesthetic, with utility at low doses in the management of depression and chronic pain. Its mechanism of action is putatively through direct synaptic or extra-synaptic NMDA receptor inhibition targeting the GRIN2B component of the NMDA receptor; inhibiting NMDA receptors localized on GABA interneurons; and activating AMPA receptors. The downstream steps for ketamine’s antidepressant effect hinge on its property of increasing neuroplasticity via EF2, BDNF, mTOR, and GSK3beta activity (Zanos and Gould, 2018). While the ketamine anesthetic dose is 2 mg/kg, the first use of ketamine as an antidepressant had positive effects at 0.5 mg/kg (Berman et al., 2000). In these early trials results were noted at 2 h after infusion and lasting past 7 days in treatment-resistant depression (Zarate et al., 2006). In 2013, a clinical trial of ketamine in treatment-resistant depression used midazolam as an ‘active’ placebo, finding 64% responders for ketamine and 28% for midazolam (Murrough et al., 2013), while the antidepressant effect was also confirmed to occur in bipolar depression (Diazgranados et al., 2010).

NMDA inhibitors such as phencyclidine (PCP) were identified in the 1980s, and noted to cause psychosis (Berry et al., 1983), leading to the development of the NMDA ‘hypofunction’ model of schizophrenia (Olney et al., 1999). Finding potential use for NMDA antagonists as anesthetic agents, PCP was introduced for this use but had untoward side effects, and was quickly replaced by the non-competitive NMDA antagonist, ketamine. In fact, PCP was noted to induce vacuolar formations in cingulate and retrosplenial cortex associated with the induced psychosis if used for brief periods (Olney et al., 1989), while more prolonged use resulted in neuronal degeneration in cerebro-cortical and limbic brain regions (Corso et al., 1997). The notion that NMDA receptor-driven glutamatergic tone controls inhibitory output for release of glutamate, acetylcholine, serotonin and norepinephrine via NMDA receptors in GABA interneurons may explain how NMDA antagonists can cause a toxic psychosis-inducing neurotransmitter surge. Additionally, NMDA receptors located preferentially on fast-spiking GABA interneurons, can be blocked by ketamine causing a late disinhibitory effect on pyramidal neurons and ensuing increased cortical excitability (Seamans, 2008). The idea that ketamine causes a release of excitatory drive by pyramidal neurons through inhibition of NMDA-receptor GABA interneurons was confirmed *in vivo* by the effects of ketamine in extracellular single-unit activity recorded from medial prefrontal cortex in freely moving rats (Homayoun and Moghaddam, 2007). A more recent study in human participants using carbon-13 magnetic resonance spectroscopy (13^C-MRS) and subanesthetic doses of ketamine showed that the glutamate/glutamine ratio (synaptic strength) was increased in correlation with a measure of the dissociative state, suggesting that prefrontal cortex glutamate release follows use of ketamine (Abdallah et al., 2018). In this context the use of ketamine continues to be an area of research for efficacy and safety for treating acute mood states and suicidality.

Lower doses of ketamine, on the other hand, have been used in models of chronic pain which highlight the role of PKC in modulating the adaptive response of the organism to nociception. A chronic pain animal model consisting of a constriction injury of the sciatic nerve in rats produces an increase in CNS excitatory amino acids (l-glutamate, l-aspartate), an activation of the pkc cascade and an increase in nitrous oxide (NO) (Mayer et al., 1999). The pkc cascade appears to play a role in augmenting pain signaling in this context. Another model of hyperalgesia is the mu opioid receptor agonist-induced hyperalgesia via stimulation cAMP by PLC and the phosphoinositide signaling cascade which specifically increases prkcg activity (Park et al., 1993). As with the sciatic nerve constriction model, an increase in prkcg may modulate opioid-signaling efficacy by triggering the activation of NMDA receptors, recruiting pain signaling systems. Notably, prkcg is known to phosphorylate the NMDA receptor one subunit at serine position 890 (Sánchez-Pérez and Felipo, 2005). On the other hand, use of NMDA antagonists decrease opioid-induced hyperalgesia (Célèrier et al., 1999). In a confirmatory experiment, Galeotti et al., examined the pkc isoforms which are involved in a thermal hyperalgesia model, finding that a PLC/prkcg/NMDA pathway constituted the nociceptive pathway, while the NMDA antagonist MK-801 and ketamine abolished hyperalgesia (Galeotti et al., 2006). While this models speaks to the peripheral effect of prkcg manipulation in the area of spinal cord Lamina II, a prkcg-dense area responsible for receiving sensory-related stimuli, including noxious, non-noxious stimuli, and regulation of pain sensations (Merighi, 2018), it is still unclear whether prkcg downregulation by ketamine plays a role in its central effects, although the potential for this effect exists in theory.

The effects of ketamine on prkcg have been observed in several experiments related to the apoptotic potential of ketamine. Ketamine administered for 3 days at higher doses increases apoptosis of rat hippocampal CA1 and dentate gyrus cells by suppressing phosphorylated-prkcg, phosphorylated-ERK1/2 and Bcl-2 expression and at postnatal day 7, high dose ketamine produced learning and memory impairments into adulthood (Galeotti et al., 2006). This induction of apoptosis is mediated by a microRNA, mir-34c, associated with neurodegenerative disorders (Zovoilis et al., 2011). In this model, genetically knocking down mir-34c, which is increased by ketamine, is accompanied by increased activity of pkc (Cao et al., 2015). In fact, antagonism of NMDA receptors by ketamine induces early and late apoptosis of cultured hippocampus neurons by inhibiting pkc and erk; the opposite, activation of NMDA receptors reverses the inhibitory effect of ketamine on pkc and erk (Jiang et al., 2018). The inhibitory effect of ketamine on prkcg in promoting apoptosis provides further grounds for more extensively examining the more general effects of ketamine in relation to prkcg, in order to further illuminate potential ketamine mechanisms of action.

### 2.7 Neuromodulation

#### 2.7.1 Electroconvulsive therapy

A single electroconvulsive seizure in rats induces increases in the phosphorylation level of pkc substrates GAP-34, MARCKS and neurogranin suggesting significant synaptic remodeling induced by the seizure. In a study of the effect of ECT on targeted pkc substrates, an acute increase in pkc activity in a comparable manner to the acute effect of antidepressants was elicited. Whether “chronic” use of ECT induces subsequent downregulation of PKC activity, like antidepressants, has not been investigated (Kim et al., 2010).

### 2.8 Future directions

A surprising group of pharmacological agents that are currently widely used in the treatment of psychiatric conditions have the property of negatively modulating pkc and in particular, prkcg (Table 1). These contemporary observations complete a circle of research which started with the initial investigations into the mechanisms of learning and memory implicating pka and pkc (Ghirardi et al., 1992; Zaheer and Lim, 1997), while also including more contemporaneous work (Breitinger et al., 2018). The examination of prkcg as a target for downregulation by pharmacological agents appears to be a plausible strategy for discovery. This approach is supported by several key observations: 1) prkcg is practically exclusive to CNS and peripheral nervous system; 2) prkcg is a key protein in the maintenance of LTP, the mechanism underlying memory and learning; 3) prkcg is stress-sensitive, with acute upregulation of prkcg causing cognitive and behavioral changes (Birnbaum et al., 2004), while a possible adaptive response of prkcg downregulation is seen in offspring (F2) of stress-affected sires (Bohacek et al., 2015); 4) prkcg modulates multiple pathways which impact neuroplasticity mechanisms (via PKA, NMDA receptors, AMPA receptors, FAK, CamKII, CREB); 5) the initial effect of multiple effective psychotropic agents (fluoxetine, lithium, valproate among others) is to acutely (1–3 days) stimulate prkcg activity, and thereafter downregulate prkcg (14–21 days). These considerations strongly point to illness-related PRCKG over-activity requiring downregulation for the effective action of the psychotropics as discussed above. In theory, gain-of-function mutations of *PRKCG* might predispose to psychiatric illness. In more extreme instances of gain-of-function mutation-induced *PRCKG* autoinhibition deficits (Pilo et al., 2022), the phenotype is spinocerebellar ataxia (SCA), due to modifications of the regulatory C1 domain (Wong et al., 2018; Shimobayashi and Kapfhammer, 2021), but less drastic and more common yet to be uncovered gain-of-function mutations may be relevant to psychiatric conditions including anxiety disorders, OCD and manic-depressive illness among others. Finally, an unexplored area of psychiatric illness in relation to PRKCG expression is sexual dimorphism, as PKC expression is up to 7-fold higher in females than in male rat hepatocytes (Zangar et al., 1995), and in rat myocytes, female sarcolemmal protective molecules are found in excess to that of male rat myocytes (Edwards et al., 2009). This observation may have implications for psychotropic mechanism of action and treatment response difference between males and females, providing a window into the mechanism of PRKCG in relation to psychiatric illness. These approaches may also gain from cross-disorder phenotype perspectives and a broader understanding of the influence of neuroplasticity in the mechanisms of illness. Future research into the modulation of PRKCG by psychotropic agents will further expand the array of options for patients in need of novel approaches to treatment-resistant psychiatric illness.

**TABLE 1:** Drugs with PKC Inhibition Properties Inducing Neuroplasticity and their Psychiatric Use.

Drug	Drug class	Mechanism	Primary Use(s)
Fluoxetine (Mann et al., 1995; Malberg et al., 2000; Encinas et al., 2006; Wang et al., 2008; David et al., 2010; Keeler et al., 2015; Umemori et al., 2018; Higuchi and Arakawa, 2023)	Serotonin Re-uptake Inhibitor	Induced-plasticity (iPlasticity)	Depression, OCD, anxiety
Tricyclics (Nalepa and Vetulani, 1991; Wong et al., 1993; Morishita and Watanabe, 1997; Dwivedi et al., 2002; Tai et al., 2007; Galeotti and Ghelardini, 2011)	Antidepressant	Decreases PRKCG activity	Depression, panic disorder
St. John’s Wort (Takahashi et al., 1989; Agostinis et al., 1995; Utsumi et al., 1995; Galeotti et al., 2010)	Natural product	Blocks PRKCG activity	Depression, anxiety
Lithium (Manji and Lenox, 1994; Manji et al., 1999; Chen et al., 2000; Wang et al., 2001; Catapano and Manji, 2008; Pandey et al., 2020; Grauffel et al., 2021)	Mood stabilizer	Substitutes Ca++ for Li+ and inhibits PKC activity	Bipolar disorder
Valproate (Watson et al., 1998; Watterson et al., 2002; Wu and Wang, 2002; Tokuoka et al., 2008)	Mood stabilizer	Increases dendritic growth	Bipolar disorder
Riluzole (Noh et al., 2000; Lamanauskas and Nistri, 2008; Lazarevic et al., 2018)	Glutamate Modulator	Inhibits PKC at catalytic site	Amyotrophic lateral sclerosis (ALS)
Tamoxifen (O’Brian et al., 1985; Radin and Patel, 2016; Gundimeda et al., 1996; Nelson et al., 2019; Lien et al., 1991; Kuhl, 2005; Novick et al., 2020; Paige et al., 1999; Einat et al., 2007; Sabioni et al., 2008; Cechinel-Recco et al., 2012; Abrial et al., 2013)	Estrogen modulator	Downregulation of PKC	Bipolar disorder
N-Acetyl-Cysteine (NAC) (Li et al., 2016; Liu et al., 2017)	Antioxidant	Decreases PRKCG phosphorylation	Skin picking, adjunct for OCD
Myricitrin (Gamet-Payrastre et al., 1999; Meotti et al., 2006; Pereira et al., 2011)	Antioxidant	Flavonoid antioxidants by inhibition of PKC	Anxiolytic, possibly anti-psychotic
Chelerythrine (Galeotti and Ghelardini, 2011; Abrial et al., 2014)	Alkaloid	Catalytic site of PKC; antidepressant PRKCG	None
Ketamine (Mayer et al., 1999; Galeotti et al., 2006; Huang et al., 2012)	NMDA antagonist	Apoptosis CA1 dentate gyrus, inhibits PRKCG	Major depression with suicidal ideation

OCD: obsessive-compulsive disorder.
